# Analyzing motivating functions of consumer behavior: Evidence from attention and neural responses to choices and consumption

**DOI:** 10.3389/fpsyg.2023.1053528

**Published:** 2023-02-09

**Authors:** Sanchit Pawar, Asle Fagerstrøm, Valdimar Sigurdsson, Erik Arntzen

**Affiliations:** ^1^School of Economics, Innovation and Technology, Kristiania University College, Oslo, Norway; ^2^Department of Business Administration, Reykjavik University, Reykjavik, Iceland; ^3^Department of Behavioural Sciences, Oslo Metropolitan University, Oslo, Norway

**Keywords:** consumer behavior analysis, motivating function, eye-tracking, frontal EEG asymmetry, motivating operations

## Abstract

Academia and business have shown an increased interest in using neurophysiological methods, such as eye-tracking and electroencephalography (EEG), to assess consumer motivation. The current research contributes to this literature by verifying whether these methods can predict the effects of antecedent events as motivating functions of attention, neural responses, choice, and consumption. Antecedent motivational factors are discussed, with a specific focus on deprivation as such a situational factor. Thirty-two participants were randomly assigned to the experimental and control conditions. Water deprivation of 11–12 h was used as an establishing operation to increase the reinforcing effectiveness of water. We designed three experimental sessions to capture the complexity of the relationship between antecedents and consumer behavior. Experimental manipulations in session 1 established the effectiveness of water for the experimental group and abolished it for the control group. Results from session 2 show that participants in the experimental group had significantly higher average fixation duration for the image of water. Their frontal asymmetry did not provide significant evidence of greater left frontal activation toward the water image. Session 3 demonstrated that choice and consumption behavior of the relevant reinforcer was significantly higher for participants in the experimental group. These early findings highlight the potential application of a multi-method approach using neurophysiological tools in consumer research, which provides a comprehensive picture of the functional relationship between motivating events, behavior (attention, neural responses, choice, and consumption), and consequences.

## 1. Introduction

Neurophysiological methods encompass a specific category of tools that can measure psychophysiological responses (e.g., using eye-tracking or galvanic skin response) and neural responses (e.g., using functional magnetic resonance imaging or electroencephalography) (see Dimoka et al., 2012 for a more detailed description of neurophysiological tools). A host of large companies, like Microsoft, Disney, Philips, and Daimler-Chrysler, have examined the application of neurophysiological tools to gain insight into consumer behavior for the benefit of their businesses (Riedl et al., 2010; Singer, 2010). Other companies, like Frito-Lay, Yahoo, and PayPal, have used neurophysiological methods to test their advertisements (Burkitt, 2009). Major broadcasting companies, like Warner Bros, CBS, Time Warner, MTV, ESPN, Fox Sports, and CNN, have employed neurophysiological research to optimize TV shows, promotional campaigns, advertising spaces, social TV, and multi-screening viewing experiences (Crespo-Pereira et al., 2019). Based on these trends, we expect to see increased usage of neurophysiological methods for consumer research in the future. Thus, the main objective of this study is to provide a methodological procedure that can provide an experimental framework for future neurophysiological consumer motivation research.

The proposed benefits of research using neurophysiological tools are that such research is less susceptible to biases and other influences (e.g., social desirability, limitations of introspection, etc.) that arise when using methods that rely on a subjective valuation (Fehse et al., 2017; Meyerding and Merz, 2018; Baumgartl et al., 2020). Thus, the growing appreciation of neurophysiological tools is linked to the search for more objective and reliable insights into consumer behavior (Nevid, 2010; Shaw and Bagozzi, 2018). An increasing number of papers have theoretically discussed the application of neurophysiological tools in related fields, such consumer research (e.g., Plassmann et al., 2007; Kenning and Plassmann, 2008; Smidts et al., 2014; Plassmann et al., 2015; Karmarkar and Plassmann, 2017; Verhulst et al., 2019). However, empirical research has not been able to keep pace with the number of conceptual publications. This paper aims to address that gap by providing a detailed, well-defined procedure that can be used to apply neurophysiological methods in conjunction with conventional experimental methods to study motivating functions of consumer behavior. Such methodological approaches that use multiple methods are more promising regarding the measurement of the influence of the transient, dynamic nature of situational determinants. Thus, the major goal of this paper is to contribute to a fuller picture of the functional relationship between antecedent events that have a motivating function on consumer behaviors, and how to study them by capturing their complexity using multiple methods. The concept of motivating operations (Laraway et al., 2003) is used to conceptualize and empirically assess the relationship between situational antecedent events and motivating functions of consumer behaviors. The aim of this paper is to capture the complexity of antecedent events that have motivating functions of consumer behaviors by studying the functional relationship between (a) motivational antecedent events, (b) consumer behavior (attention, neural responses, choice, and consumption), and (c) resulting consequences.

## 2. Literature review

### 2.1. The impact of antecedent motivational events on consumer behavior

The motivating influence of situational factors can be studied using the concept of *motivating operations*, typically used to refer to events that have motivating functions. Motivating operations are defined as an environmental event that (a) establishes (or abolishes) the reinforcing or punishing effect of another event (the value-altering effect) and (b) evokes (or abates) behaviors related to that event (the behavior-altering effect) (Laraway et al., 2003). In simple terms, a motivating operation changes how much a consumer “wants” something in a purchasing situation and how hard they will “work” to get it (Fagerstrøm et al., 2010). Motivating operations encompass two types of value-altering effects: establishing operations (EOs) and abolishing operations (AOs). EOs make consequences more effective, whereas AOs make them less effective (Laraway et al., 2014). Thus, deprivation tends to be an EO for food and water consumption, and satiation functions as an AO (Tapper, 2005). For example, water is established as an effective reinforcer after a period of water deprivation. In this situation, an organism is more likely to exhibit choice and consumption behaviors that have previously been associated with drinking. When a large quantity of water has been consumed, the effectiveness of water as a reinforcer is abolished, and simultaneously, the likelihood of previously mentioned behaviors being emitted is abated. In a choice situation where a consumer orders food and/or drinks, it is highly likely that this person is in a state of food/water deprivation. In such situations, deprivation has a motivating function (EO), and most probably influences the choice of product category and/or quantity.

The concept of motivating operations has made important contributions to both conceptual and applied research (e.g., Iwata et al., 2000; Langthorne et al., 2007; Rispoli et al., 2011; Simó-Pinatella et al., 2013; Laraway et al., 2014; Maraccini et al., 2016). The concept has also been shown to be a comprehensive framework for consumer behavior analysis in general (Fagerstrøm et al., 2010), and in particular for studying consumer online purchasing behavior (Fagerstrøm et al., 2010; Fagerstrøm and Ghinea, 2011; Fagerstrøm and Arntzen, 2013), the impact of corporate social responsibility (Fagerstrøm et al., 2015), and online hotel booking (Eriksson and Fagerstrøm, 2017). However, to the best of our knowledge, no study has tried to capture the complexity of antecedent events that have motivating functions of consumer behaviors by studying the functional relationship between antecedent events (e.g., deprivation), and different types of consumer behaviors (attention, neural responses, choice, and consumption).

### 2.2. Deprivation as a motivating antecedent event

The value-altering effect of motivating operations modifies the ability of consequences (reinforcers and punishers) to change consumer behavior. This ability is usually influenced by manipulating the associated EOs and AOs. Sundberg (1993) highlights that, in general, the reinforcing effectiveness of an EO is transient, and to be used as an independent variable, researchers can either (1) take advantage of an EO as it occurs naturally in the environment or (2) manipulate some event that alters the value of another event as a form of reinforcement. Most laboratory studies that take advantage of EOs usually involve a history of deprivation of some event that functions as primary reinforcement (Pierce and Cheney, 2013).

Deprivation is an EO that occurs naturally as a function of time. Accordingly, such studies alter the effectiveness of reinforcers by manipulating the EO of deprivation. Researchers studying the effects of deprivation typically use the first strategy (taking advantage of an EO as it occurs naturally in the environment) and manipulate the length of time since consumption of the unconditioned reinforcer. For example, Coate (1964) manipulated various lengths of water deprivation on 32 rats. Overall, the result of this study demonstrated that the strength of responding (behavior-altering effect) was directly related to the level of deprivation at the time of testing. For human participants, *ad libitum* feeding and drinking rarely allow for individuals to go for more than 24 h of food or water deprivation. Some previous research has used 16 h of water deprivation with human participants (Hallschmid et al., 2001). However, such periods of deprivation do not mimic natural settings. Forzano et al. (2010) used a procedure in which participants were scheduled to start experimentation approximately 4 h (plus or minus an hour) after their normal awakening time (without consuming any foods or liquids), resulting in a deprivation period of roughly 11–12 h. This period of deprivation mimics instances where the individual skips or delays the consumption of food or drinks in the morning. The use of such a manipulation strategy is more ecologically sound and allows for a relatively strong EO without causing discomfort to the participant.

The second strategy, that is, to manipulate some event that alters the value of another event as a form of reinforcement (the value-altering effect), is related to EOs (other than deprivation) that have established the effectiveness of a reinforcer in a similar fashion. For example, an event like ingestion of salt rich food, is not the same as a period of water deprivation but will have similar effects because it functions as an EO for water. This strategy aims to induce effects similar to water deprivation by using events that function as EOs for water. Michael (1982) clearly distinguishes between deprivation as an EO and other events that have the same effect. For example, events like ingestion of salty food, a dry climate, high temperatures, perspiration, or strenuous physical exercise may function as EOs for water and simultaneously evoke behaviors that have provided access to water in the past. These events have much the same effect as deprivation: water becomes more effective as a form of reinforcement, and behavior that has been reinforced with water becomes momentarily more frequent (Michael, 1993). In research, a consumption of a variety of salty food items has been used as a strategy to increase the value of liquids (e.g., Aarts et al., 2001; Ferguson and Bargh, 2004; Shalev, 2014). Physiologically, the addition (through ingestion) or loss (through excretion, salivary loss, respiration, perspiration, etc.) of either water or sodium to/from the body alters the net fluid balance within the body, but also the concentration of salt molecules that draw water in and out of the cell compartments (Johnson and Thunhorst, 1997). In simple terms, the concentrations of sodium and water help regulate the amount of water in the cell compartments, causing water to either leave the cell (the cell shrinks) or enter the cell (the cell expands). The balance of salt and water in the body is heavily interconnected. This is one of the main reasons why the ingestion of salt has a similar EO as water deprivation. Therefore, a mouthful of salty food can have the same effect as a period of water deprivation (Catania, 1998).

The behavior-altering effect subsumes two effects: the evocative effect and the abative effect (Laraway et al., 2003). As their names imply, these effects refer to the respective increases and decreases in the current strength of behavior influenced by motivating operations (Laraway et al., 2014). It is highly likely that many consumer behaviors are affected by a given motivating operation (Laraway et al., 2003). Deprivation, as a motivating operation, has been shown to cause changes in attention (Castellanos et al., 2009), neural responses (Nijs et al., 2010), and behavioral tendencies (Hoefling and Strack, 2008). By focusing on deprivation states and their motivating functions, we can improve our understanding of how such antecedent events in their entirety influence consumer behavior. These will be discussed in more detail in the sections that follow.

### 2.3. Attention

Research provides evidence that motivationally relevant stimuli significantly impact visual behavior (West et al., 2009). Most of the reported events in eye-tracking data relate to fixations, which are defined as the state when the eye remains relatively still over some time (Holmqvist et al., 2011). During a fixation, a small area of the visual field is projected onto the fovea (an area of the eye with superior visual acuity) for detailed visual processing (Wedel and Pieters, 2008; Chandon et al., 2009). Eye fixations, used as a measurement of attention, forms the basis of many measures and metrics used in consumer research. These measures relate to the number of fixations (e.g., fixation count) and the length of fixations (e.g., first/last/average/total fixation duration) (see Holmqvist et al., 2011 for a more detailed overview of fixation metrics). Fixation count indicates how many times the participant looks at the area of interest (AOI) (Behe et al., 2015). Metrics that measure eye fixation duration reflect the amount of attention given to an area of interest and vary based on the type of stimuli (e.g., texts or graphics) and/or types of tasks (e.g., reading or problem-solving) (Tsai et al., 2012). Most of the measures of visual attention used in eye-tracking research are adept at predicting product choices (Russo and Leclerc, 1994; Pieters and Warlop, 1999; Chandon et al., 2006; Wedel and Pieters, 2007). For example, consumer research on brands has shown that longer average fixation durations are positively correlated with choice (Pieters and Warlop, 1999). In food research, fixation count has shown to be positively correlated with food choice (Jantathai et al., 2013). Taken together, these findings demonstrate the importance of visual attention for choice behavior, and can thus be used to predict eventual choice behavior in-store. By examining both metrics, we can discern which measure of attention, related to consumer choice, can be linked to motivating operations.

### 2.4. Neural responses

Modern advances in neurophysiological tools have allowed for a more detailed analysis of consumer behavior. Neuroscientific research utilizes technologies that allow measurement of responses undetected by tools that have traditionally been used by behavior analysts (Ortu, 2012). This is because neurophysiological tools can measure responses that often precede overt behavioral responses (Palmer, 2010). One such tool is an EEG, which measures the changes in the brain’s electrical activity through external electrodes placed on the scalp. These electrodes measure the synchronized electrical activity of vertically aligned pyramidal cells in the neocortex (Teplan, 2002). The resulting electrical signal is a mixture of several base frequencies captured by an electrode. These frequency ranges (or bands) are classified as delta (1–4 Hz), theta (4–8 Hz), alpha (8–12 Hz), and beta (12–25 Hz) (Khushaba et al., 2013).

A substantial amount of research has suggested that electrical neural activity in the frontal regions of the brain is linked to motivation (Harmon-Jones et al., 2010; Harmon-Jones and Gable, 2018). This measure of the frequency-based electrical differences between the left and right hemispheres in the frontal brain regions is termed *frontal EEG asymmetry*. More specifically, the relative difference in alpha power (activity of the alpha band frequency) between the right and left frontal cortical regions is measured (Allen and Reznik, 2015). Frontal EEG asymmetry differences are calculated as an index: log (alpha EEG power right F4) minus log (alpha EEG power left F3) (Allen et al., 2004). Frontal EEG asymmetry has often been cited as a useful measure to explain and predict behavior (Jackson et al., 2003; Rosenkranz et al., 2003; Goodman et al., 2013).

Substantial research supports the concept that frontal asymmetry is correlated to two proposed *neural-behavioral systems* associated with an approach system and a withdrawal system (Davidson, 1998, 2004). According to this concept, frontal EEG asymmetry corresponds to motivational or behavioral tendencies to approach versus withdraw. Specifically, approach-related tendencies are reflected by the relatively greater activity in the left frontal cortex and the converse is true for withdrawal-related tendencies (Tomarken et al., 1990; Davidson, 1992, 1998, 2003; Demaree et al., 2005; Harmon-Jones et al., 2010). Further research suggests that in humans, these asymmetric activations are often specific to the frontal cortex, and activity in one hemisphere inhibits the other (Harmon-Jones et al., 2010).

Unconditioned biological processes have an EO effect (i.e., appetitive/approach motivation) and evoke approach-related behaviors that have led to access to those reinforcers in the past. These unconditioned motivating operations mainly include deprivation and satiation of unconditioned reinforcers like food and water (Laraway et al., 2014). Frontal EEG asymmetry can be used to measure the effects of deprivation. For example, in a study by Zinser et al. (1999), the authors used frontal EEG asymmetry to examine smoking motivation in relation to tobacco deprivation and exposure to smoking cues. They found that cigarette cues elicited increased asymmetry, while actual smoking did not. This result is highly similar to the effects of motivating operations discussed previously. The period of deprivation and presentation of smoking cues changed the reinforcing value of tobacco. When the reinforcer was obtained, the effectiveness of smoking was abolished. Another study where food deprivation was manipulated demonstrated that time since eating and self-reported liking for dessert were associated with greater relative left frontal EEG activation (approach motivation) during the viewing of dessert pictures, but not during the viewing of neutral pictures (Gable and Harmon-Jones, 2008; Harmon-Jones and Gable, 2009). Similarly, exposure to alcohol cues by individuals who reported liking alcohol has shown greater relative left frontal EEG activation when compared to neutral images (Gable et al., 2016). Taken together, these results suggest that frontal EEG asymmetry can be used to verify motivating functions of deprivation at a neural level.

### 2.5. Choice and water consumption

Consumer choice and consumption are relatively complex behaviors that include many responses. Each choice the consumer makes is influenced by an antecedent event which also has motivating functions related to the final choice. A state of water deprivation, which is highly relevant in a foodservice, convenience, or grocery store when buying something to drink, will most probably have an EO effect on beverages and simultaneously evoke choices of products within that category.

### 2.6. Aims of the current study

In general, studies have shown that food deprivation leads to increased attention and reactivity toward food cues (Lavy and van den Hout, 1993; Mogg et al., 1998; Stockburger et al., 2009). Similarly, deprivation has been shown to cause changes in eye-movement behavior. Research by Castellanos et al. (2009) has demonstrated that eye-movement behavior among food-deprived participants is significantly inclined toward food images (when compared to non-food images), in terms of gaze duration and direction. Recent reviews suggest that such influences on attention are unrelated to individual differences in body weight (Hardman et al., 2021). Given the evidence, in a situation where a consumer is water-deprived, it is reasonable to assume eye-tracking can be used in verifying motivating functions in relation to attention toward beverages. Deprivation of water has an EO effect on beverages (the value-altering effect), and secondly, it evokes eye-movement behaviors related to beverages (the behavior-altering effect).

As mentioned previously, evidence suggests that frontal EEG asymmetry holds potential for measuring unconditioned motivating operation effects like deprivation. Hence, frontal EEG asymmetry can be used in verifying motivating functions when a consumer is water-deprived.

It is rare in studies related to consumer behavior to investigate actual consumption. The behavior-altering effect of motivating operations encompasses all responses, from search, choice, and final consumption. Thus, to capture the complexity of consumer behavior, we decided to include consumption. A state of water deprivation will most probably have an EO effect on beverages and simultaneously evoke choices of products within that category.

We asked four related research questions, namely, will deprivation have an EO effect on beverages and evoke: (1) a higher fixation count and longer average fixation duration toward the relevant reinforcer?; (2) relatively greater left frontal activity (approach motivation) toward the relevant reinforcer?; (3) choices of the relevant reinforcer?; and (4) greater consumption of the reinforcer? Furthermore, the prediction is that satiation (having an AO effect) will not be shown to have the same impact as that asked in the four research questions.

## 3. Method

### 3.1. Participants

Initially, 39 students and faculty members agreed to participate in the study which comprised of three sessions. Four participants failed to comply with the deprivation procedure and were removed. Two participants were removed due to invalid cases. The sample was restricted to right-handed participants. The use of homogeneous samples related to handedness affects the validity of EEG experiments because hemispheric asymmetry is strongly connected to hand dominance (Davidson et al., 1990). Consequently, hemispheric specialization is strengthened by the consistent use of the dominant/preferred hand. Handedness was assessed by the Chapman Handedness Inventory (Chapman and Chapman, 1987). One participant was categorized as ambidextrous (scored 28 on the handedness inventory) and was removed from the analysis. Any personal identifiable information was not collected.

The final sample comprised of a total of 32 (18 male, 14 female) right-handed [scored ≤17 on the Chapman and Chapman (1987) handedness inventory], normal-weight adults ranging in age between 19 and 37 years (*M* = 24.2; *SD* = 3.54). All participants received 200 NOK (approximately 20 USD) compensation in exchange for participation. The participants were recruited using flyers and class presentations. The recruitment was done under the pretense that the researcher was measuring the influence of taste using neurophysiology. The main reason for doing so was to introduce the relatively unfamiliar EEG and eye-tracking data collection methods. Additionally, the cover story allowed masking of the water deprivation manipulation (influence of hydrated state versus dehydrated state on taste), minimizing any possible demand effects. Participants were informed that the experimental procedure would take anywhere between 45 min and an hour and were randomly assigned to the experimental (water deprivation) group and control group. Written consent was obtained from all participants using an onscreen consent form. The participants were randomly allocated to each group and were given instructions accordingly. The experimental group consisted of 10 males and 6 females. The control group consisted of eight males and eight females. Participants in both groups took part in all experimental sessions. Upon arrival, the participants were given a brief overview of the three experimental sessions. Procedural instructions for all sessions were provided in written format onscreen. The use of smaller sessions allowed the participants to take small breaks (if needed) between the experimental proceedings. They were informed that they could quit any time if they wanted to. At the end of the experimental sessions, participants were asked what they thought the purpose of the study was. None of the participants were able to guess the main purpose of the study.

### 3.2. Overview of experimental sessions

We conducted three experimental sessions. In session 1, we wanted to further establish the effectiveness of a reinforcer for the experimental group and abolish it for the control group. The participants in the experimental group had to consume salty biscuits and participants in the control group had to consume flavored water. The session demonstrated that there were differences in the motivating function of water deprivation and satiation for the two groups in terms of thirst. In session 2, we wanted to collect eye-tracking metrics and frontal asymmetry index values for participants in the two groups. The participant viewed a series of neutral images (required for baseline corrections in EEG data) and images of salty snacks and liquid beverages. The images of interest were the image of the salty biscuits and glass of water. This session demonstrated some differences for eye-tracking metrics within-group and some indication of changes in frontal EEG alpha asymmetry index values. In session 3, we stimulate choice through paired choice trials using images of salty snacks against liquid beverages. Another aim of this session was to get a measure of actual consumption, thus participants were offered a 500 ml water bottle to drink at the end of the study. This session demonstrated differences in choice and consumption behaviors for the experimental group compared to the control group. The data were exported from the research platform software as a CSV file. All data were analyzed using IBM SPSS Statistics version 28.

### 3.3. Session 1: Baseline data collection and additional manipulation

Research procedures that manipulate deprivation in human subjects show various deprivation lengths of anywhere between 3 and 15 h (Strahan et al., 2002; Raynor and Epstein, 2003; Hoefling and Strack, 2010). This study employed a procedure similar to Forzano et al. (2010). Participants in the experimental group were asked to show up for data collection 3 h after their normal waking up time, resulting in a deprivation duration of 11–12 h (8 h + 3–4 h). Thus, for standardization, participants in the experimental group were instructed to refrain from drinking any liquids for 3 h (plus or minus an hour) in the morning before any data collection began. Such a situation will mimic instances where the individual postpones the consumption of the first food/drink item to a later time. Additionally, this deprivation period will effectively replicate the deprivation state that most individuals will experience in consumer settings. Since we were manipulating only the deprivation of water and not food, participants in the experimental group could eat dry solid foods during this time period. Participants in the control group could show up at any time for data collection and had no restrictions on food or water intake.

#### 3.3.1. Design

The survey questions involved thirst, handedness, mood, and tasting items. For the manipulation check, all participants were asked to report the extent to which they currently felt thirsty on a 7-point Likert scale onscreen (1 = not thirsty at all; 7 = very, very thirsty). Handedness was measured using the Chapman and Chapman (1987) inventory consisting of 13 questions about handedness. In some cases, mood can show sensitivity to changes in water deprivation/consumption (see Masento et al., 2014). Thus, we measured mood in case we needed to control for its effects. *A priori* mood differences were examined by asking the participants the following: “On a scale of 1–7, what is your current mood state?” ranging from 1 (very negative) to 7 (very positive). In addition, three questions consisting of the tasting items were introduced to lend credibility to the cover story. These questions were: (1) “What was the flavor of the biscuit?” (open-ended); (2) “How would you rate the flavor of the biscuit?” (scale: 0 = horrible to 6 = excellent); and (3) “How likely are you to recommend this to others?” (scale: 0 = not at all to 6 = certainly).

#### 3.3.2. Apparatus

For Session 1, participants in the experimental group had to taste at least four salty biscuits and the control group tasted flavored water, after which both groups answered survey questions. For the experimental group, the four flavors of salty biscuits were presented individually on disposable white paper plates, covered with a napkin and correspondingly marked 1 to 4. Each plate had three individual units (three biscuits) of the specific flavor (three flavors and one plain salted variety). For the control group, the four water samples were similarly presented in four clear disposable plastic cups covered with a napkin and correspondingly marked 1 to 4. Each cup contained 100 ml of the specific water flavor (three flavors and one neutral variety). A dual-screen setup was used, where the screens were divided by a partition. Two 24-inch widescreen monitors with a resolution of 1920 × 1,080 were used. The primary screen was placed in front of the experimenter and the secondary screen was placed in front of the participants (used to display the slideshow). The experiment was conducted using iMotions® research platform software.^1^ In this session, the participants progressed through the slideshow by clicking a centrally placed button marked “next” at the bottom of the screen using a standard mouse and keyboard.

#### 3.3.3. Procedure

This session began with the general introduction to the experiment sessions and the consent form. This was followed by the mood scale and the Handedness Inventory (Chapman and Chapman, 1987). Thereafter, the cover story was presented, which stated that the main purpose of the experiment was to examine the relationship between taste and neurophysiology. Participants in the experimental group were told that the manufacturer, Mondelez International, has introduced new varieties of salty biscuits to the market. These are rectangular salty biscuits (product name: TUC) that are widely available in Norway, and have a sodium content between 1.8 and 2.9 g per 100 g. Participants were instructed to taste the four different flavors of salty biscuits (original salty, and three other random flavors). The onscreen instructions were to taste the biscuits from the plates (in numerical order) and to click in order to proceed to the tasting questions. The participants were verbally informed that they could take their time while tasting and should completely eat at least one biscuit from each of the plates. This was repeated four times for each of the flavors. The last flavor was always the original salted version; the order of the other three flavors was randomized. The participants in the control group followed a similar procedure where they had to taste four different flavors of water (three random flavors with the last one always being regular water) and answer tasting questions. After tasting, the participants responded to the thirst scale item, followed by an information slide stating that the session was completed.

### 3.4. Analysis and results

#### 3.4.1. Mood ratings

Subjective reports of mood were examined for both groups. A Mann–Whitney *U* test revealed no *a priori* differences in mood between participants in the experimental group (Mdn = 5.00, *n* = 16) and participants in the control group (Mdn = 5.50, *n* = 16), *U* = 127.00, z = −0.04, *p* = 0.967, *r* < 0.01.

#### 3.4.2. Manipulation check

Results from a Mann–Whitney *U* demonstrated that the experimental group (Mdn = 5.50, *n* = 16) reported stronger feelings of thirst than the control group (Mdn = 4.00, *n* = 16), *U* = 24.50, z = −3.98, *p* < 0.001, with a large effect size *r* = 0.7. Therefore, the manipulations successfully established a stronger motivating operation for the experimental group than for the control group.

### 3.5. Session 2: Attention and neural responses

This session was designed specifically to study attention and neural responses by collecting eye-tracking and EEG data, respectively. The main aim of the second session was to measure the effects of water deprivation/satiation on attention and neural responses (the behavior-altering effect), linked to research questions one and two. Having a separate session for the collection of eye-tracking and EEG data allows for the minimization of any noise artifacts that might be generated from extreme physical movement. Therefore, this session was comprised entirely of viewing stimulus images as they changed onscreen.

#### 3.5.1. Design

The session consisted of a viewing condition task where both groups viewed exactly the same slideshow. The slideshow started with a series of 12 randomized neutral images (presented in succession) from the Geneva affective picture database (GAPED) (Dan-Glauser and Scherer, 2011). Each image remained onscreen for 15 s. This was done to get a relatively similar starting baseline for all the participants before the collection of eye-tracking and EEG data of interest to the study. This was followed by a second series of randomized images that consisted of five liquid item images in a clear glass (still water, sparkling water, milk, cola, and juice) and salty snacks in a clear bowl (pretzel sticks, peanuts, chips, crackers, and salty biscuits). All images were separated by a black inter-slide which remained onscreen for 20 s to minimize carry-over effects of the previous image. The rating for thirst was recorded again at the end of this session.

#### 3.5.2. Apparatus

Eye-tracking data were collected using a Tobii X2-30 screen-based eye-tracker with a sampling rate of 30 Hz. The eye-tracker was attached to the bottom of the 24-inch secondary screen used to display the slideshow. A standard mouse and keyboard were used by the participants to start and end the slideshow using iMotions research software. The EEG model used for the collection of neurophysiological data was the Advanced Brain Monitoring (ABM) B-Alert X10 headset with nine electrode channels according to the International 10/20 system (F3, Fz, F4, C3, Cz, C4, P3, POz, and P4), with a sampling rate of 256 Hz. The EEG headset was fitted to the back of the participant’s head. The participant’s head was cleaned using an alcohol-based cleaning wipe. Conductive cream was applied to all electrodes. The linked reference electrodes were placed behind each ear on the mastoid bone. Electrode impedances were kept below 20 kΩ.

#### 3.5.3. Procedure

At the beginning of this session, an impedance check was performed for the EEG. Upon reaching the acceptable level (20 kΩ), the participants performed a calibration for the eye-tracker. The participant was instructed to look at nine points on the screen (fixation locations) to calibrate the eye-tracker to his or her eyes. After calibration, the participant clicked the onscreen button to start the first neutral images slideshow and then to start the test image slideshow. The second slideshow was followed by a thirst scale for the manipulation check. The final slide informed the participants that the session was completed.

### 3.6. Analysis and results

#### 3.6.1. Thirst manipulation check

Thirst was measured again to check whether the deprivation/satiation effect was still active for the respective groups. A Mann–Whitney *U* demonstrated that the experimental group (Mdn = 6.00, *n* = 16) reported stronger feelings of thirst than the control group (Mdn = 4.00, *n* = 16), *U* = 26.00, z = −3.96, *p* < 0.001, with a large effect size *r* = 0.7. Therefore, water was still active as a reinforcer for the experimental group during Session 2. For the control group, the effectiveness of water was abolished during eye-tracking and EEG data collection.

#### 3.6.2. Eye-tracking

The stimulus images were displayed centrally with black borders around each image to allow for comfortable viewing on the 24-inch widescreen monitor. For the analysis of eye-tracking data, the AOIs were manually defined around both stimulus images (salty biscuits and water) using the built-in functionalities in the iMotions software. Eye-tracking data quality, reported by the iMotions software, provided quality assurance metrics that show the % of time the respondent looked at the screen. For the experimental group, this was at 91% for the water stimulus image and 92% for the salty biscuit stimulus image. Reported data quality for the control group was 94% for both the water and salty biscuit stimulus images. For each participant, we calculated the fixation count and the average duration of fixations within the AOIs. We compared the values within the groups because, for the experimental group, the water deprivation and salty biscuit tasting manipulation (eating something salty, increasing the effect of water deprivation) corresponded to an EO. Thus, the water would have an AO effect (on water deprivation). This means that for the experimental group, the water image will correspond to an AO and the salty biscuit image will correspond to an EO. For the control group, only the water image would have an effect, since the manipulation to drink flavored water would have an AO effect on thirst. This means that for the control group only the water image will correspond an AO while the biscuit image will correspond neither an EO nor an AO. Therefore, the eye-tracking metrics relating to the salty biscuit image and water image were compared within-group.

For each group, comparisons between the fixation count values for the salty biscuit stimulus image and water stimulus image were conducted. Shapiro–Wilk tests did not show evidence of non-normality for the experimental group fixation count datasets for the salty biscuit image *W*(16) = 0.973, *p* = 0.881 and water stimulus image *W*(16) = 0.918, *p* = 0.157. Based on this outcome, a dependent samples *t*-test was utilized. Results show significant differences in fixation count values within the experimental group for the salty biscuit image (*M* = 31.94, SD = 6.72) and water image (*M* = 26.81, SD = 6.97), *t*(15) = 3.58, *p* = 0.003 (two-tailed), Hedges’ *g* = 0.85; 95% CI = 0.29–1.39.

For the control group fixation count dataset, the Shapiro–Wilk tests provided evidence of departure from normality for the salty biscuit image *W*(16) = 0.881, *p* = 0.041 and water stimulus image *W*(16) = 0.619, *p* < 0.001. Therefore a non-parametric, Wilcoxon signed-rank test for non-parametric dependent small samples was used. The test revealed no significant differences in fixation count for the salty biscuit image (Mdn = 32.50, *n* = 16) and water image (Mdn = 29, *n* = 16), *z* = −1.35, *p* = 0.176, *r* = −0.24.

The average fixation duration was compared to ascertain differences between the water and salty biscuit images within the two groups. Shapiro–Wilk tests did not show evidence of non-normality for the experimental group average fixation duration datasets for the salty biscuit image *W*(16) = 0.97, *p* = 0.837 and water stimulus image *W*(16) = 0.959, *p* = 0.649. Based on this outcome, a dependent samples *t*-test was utilized. A statistically significant difference was evident in the average fixation duration (in milliseconds), in the experimental group for the salty biscuit image (*M* = 305.5, SD = 71.62) and water stimulus image (*M* = 351.81, SD = 118.37), *t*(15) = −2.29, *p* = 0.037 (two-tailed), Hedges’ *g* = −0.54; 95% CI = −1.04 to –0.03.

Shapiro–Wilk tests did not show evidence of non-normality for the control group average fixation duration datasets for the salty biscuit image *W*(16) = 0.941, *p* = 0.362 and water stimulus image *W*(16) = 0.984, *p* = 0.987. Therefore, a dependent samples t-test was utilized. No significant differences were found for the average fixation duration values (in milliseconds) for the control group for the salty biscuit image (*M =* 285.88, SD = 62.37) and the water image (*M* = 288.25, SD = 82.51), *t*(15) = −0.172, *p* = 0.866 (two-tailed), Hedges’ *g* = −0.04; 95% CI = −0.51 to 0.43.

#### 3.6.3. Frontal asymmetry

Neural activity was recorded from F3 and F4 electrodes on the frontal cortex. The relatively higher activation of the left hemisphere compared to the right hemisphere was used as a neural index of approach motivation (Davidson et al., 1990; Tomarken et al., 1990; Zinser et al., 1999; Davidson, 2004; Ravaja et al., 2013; Garczarek-Bąk and Disterheft, 2018).

The EEG signals for each stimulus were extracted using the stimulus on and offset timing provided by the data collection software. The raw EEG signals from the F3 and F4 electrodes were z-scored to obtain relative amplitudes of the frequencies of interest. For the stimulus images (neutral GAPED images, water, and salty biscuit), power spectral densities in the alpha frequency range (8–12 Hz) were computed for both electrodes using the NeuroSpec toolbox package v2.0^2^ for MATLAB (2020). The Power Spectral Density (PSD) estimates were carried out using methodologies set out in Halliday et al. (1995) using 1-s segments with zero overlap. The PSD estimates were then used to calculate the mean frontal alpha asymmetry (FAA) which measures the difference between distributions of frontal alpha activity across the two hemispheres. First, the log-transformed difference of the PSD estimates at each frequency (8–12 Hz) was calculated:

FAA_8-12 Hz_ = Log(F4)-Log(F3) (Calculated for each frequency).

This provided a difference for each of the frequencies between 8 and 12 Hz. As a last step, the mean of differences across the range of frequencies was calculated to obtain the measure of frontal asymmetry. In neurophysiological research, it is known that neural activation patterns are highly reliable within-subjects but not necessarily between-subjects (see Corsi-Cabrera et al., 2007). Within-subjects experimental designs can overcome issues with cortical differences by using a proper baseline for meaningful comparisons (Dimoka et al., 2012). Such designs can help reduce error variance by using each subject as her/his own control, as individuals provide their own optimal baseline for measuring activation differences (Dimoka et al., 2012; Daugherty et al., 2016). Therefore, the index scores of the 12 neutral GAPED images were averaged for each participant and the resulting score was used as the baseline for the participant. This baseline score was then subtracted from the index score of the participant for the salty biscuit image and water image. Therefore, the frontal asymmetry scores were compared within-group.

Shapiro–Wilk tests did not show evidence of non-normality for the experimental group frontal asymmetry index value datasets for the salty biscuit image *W*(16) *=* 0.977, *p* = 0.932 and water stimulus image *W*(16) = 0.987, *p* = 0.996. Based on this outcome, a dependent samples *t*-test was utilized. Results show no significant differences in the experimental group for the salty biscuit image (*M = −*0.009, *SD =* 0.59) and water image (*M =* 0.033, *SD* = 0.59), *t*(15) = 2.07, *p* = 0.056 (two-tailed), Hedges’ *g* = −0.49; 95% CI = −0.98 to 0.01.

For the control group frontal asymmetry index values dataset, the Shapiro–Wilk tests did not show evidence of non-normality for the salty biscuit image *W*(16) = 0.909, *p* = 0.112. However, the frontal asymmetry index values for the water image provided evidence of departure from normality *W*(16) = 0.882, *p* = 0.041. Therefore, a non-parametric, Wilcoxon signed-rank test for non-parametric dependent small samples was used. The test revealed no significant differences in frontal asymmetry index values for the salty biscuit image (Mdn = −0.007, *n* = 16) and water image (Mdn = 0.003, *n* = 16), *z* = −0.83, *p* = 0.41, *r* = −0.15.

### 3.7. Session 3: Choice and water consumption

The purpose of this session was to measure the behavior-altering effects of water deprivation/satiation on choice and actual consumption, linked to research questions three and four.

#### 3.7.1. Design

The third session consisted of paired choice comparisons. To simulate choice (clicking behavior), the last session consisted of 25 paired choice trials (5 liquids × 5 food items). The liquid beverage and salty snack images from the second session were used again. In each trial, participants were instructed to choose between two options consisting of a liquid beverage and a salty snack. The position of each option on the screen (right versus left side) was counterbalanced between the pairings. The order of the trials was randomized. Finally, post-experimental questionnaires recorded demographic information and a question on hypothesis guessing. The participants were then informed that they were “free to drink water” and were given a 500 ml bottle of water and a plastic cup. The remaining water left in the 500 ml bottle was used to measure the actual consumption of water for participants in both groups.

#### 3.7.2. Apparatus

In this session, participants in both groups had to choose between the two options presented onscreen, consume water, and answer demographic information. Using a standard mouse and keyboard, all participants completed two-alternative forced-choice questions and demographic information. A 500 ml bottle of water and a disposable plastic cup were given to the participant at the end of the session for consumption. A standard digital food scale was used to measure the remaining water in the bottle (after consumption).

#### 3.7.3. Procedure

The participants started the slideshow and answered the 25 paired choice trials (5 liquids × 5 food items). In each trial, participants were instructed to choose between two options. One of these options always corresponded to a liquid beverage (still water, sparkling water, milk, cola, and juice); the other option always corresponded to a salty snack (pretzel sticks, peanuts, chips, crackers, and salty biscuits). Thereafter, the participants answered the demographic questions onscreen. At the end of the session, participants consumed water from the bottle provided using the plastic glass.

### 3.8. Analysis and results

#### 3.8.1. Paired choice comparisons

**Figure 1:**
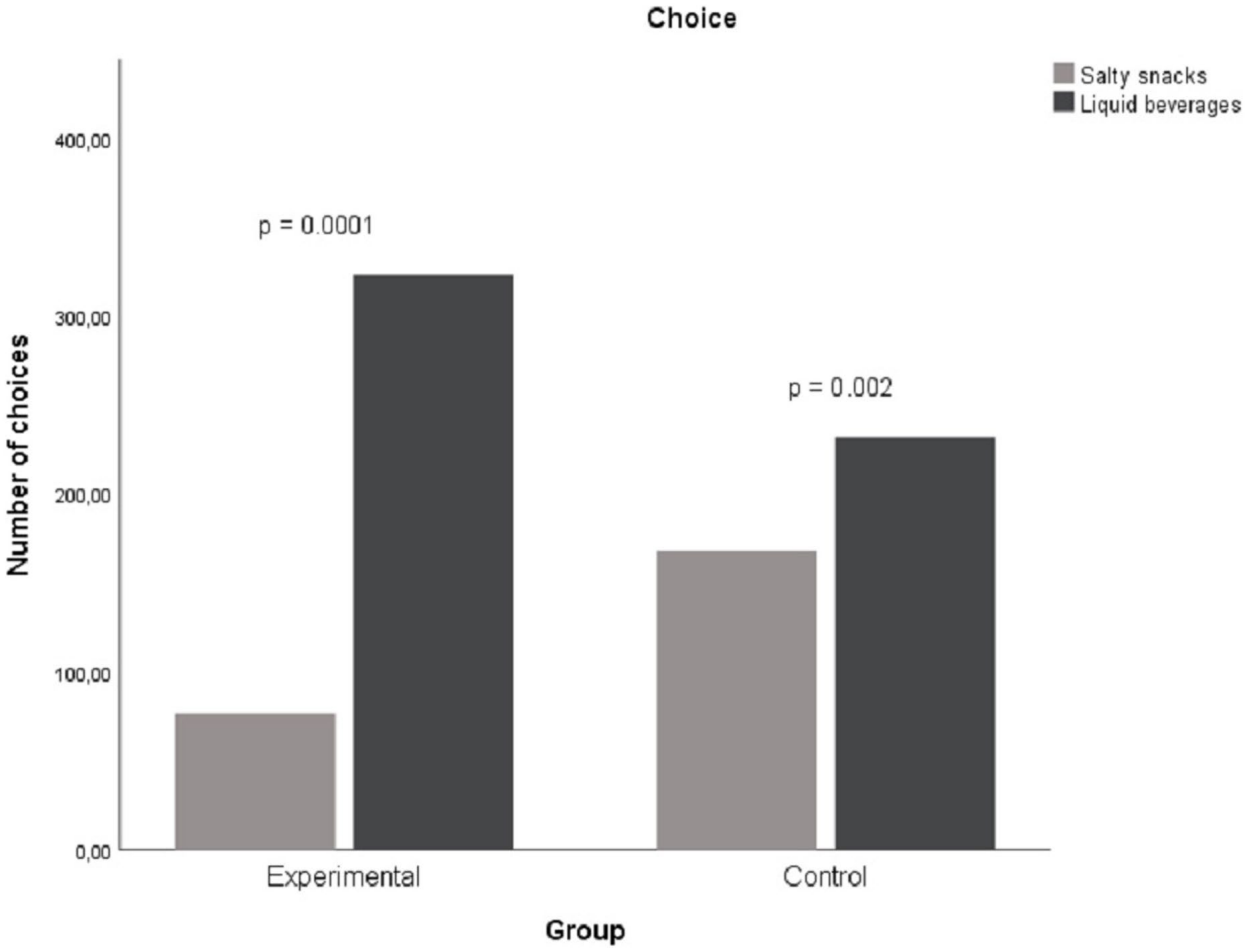
Difference in choice of reinforces in session 3.

Figure 1 below shows choice differences between groups. We tested whether choice proportions differed between the groups when choosing either a salty snack or a cold beverage for the paired choice comparisons. Liquid beverages were chosen more often by participants in the experimental group (81%; 323/400) compared to the control group (58%; 232/400). A Chi-square test of independence was performed to evaluate the relationship between group and choice of liquid beverage. The results of Chi-square test of independence (with Yates Continuity Correction) revealed that relationship between these variables was significant *X*^2^(1, *N* = 800) = 47.66, *p* < 0.001, phi = 0.28. Participants in the experimental group were more likely to select liquid beverages than participants in the control group.

#### 3.8.2. Amount of water consumed

**Figure 2:**
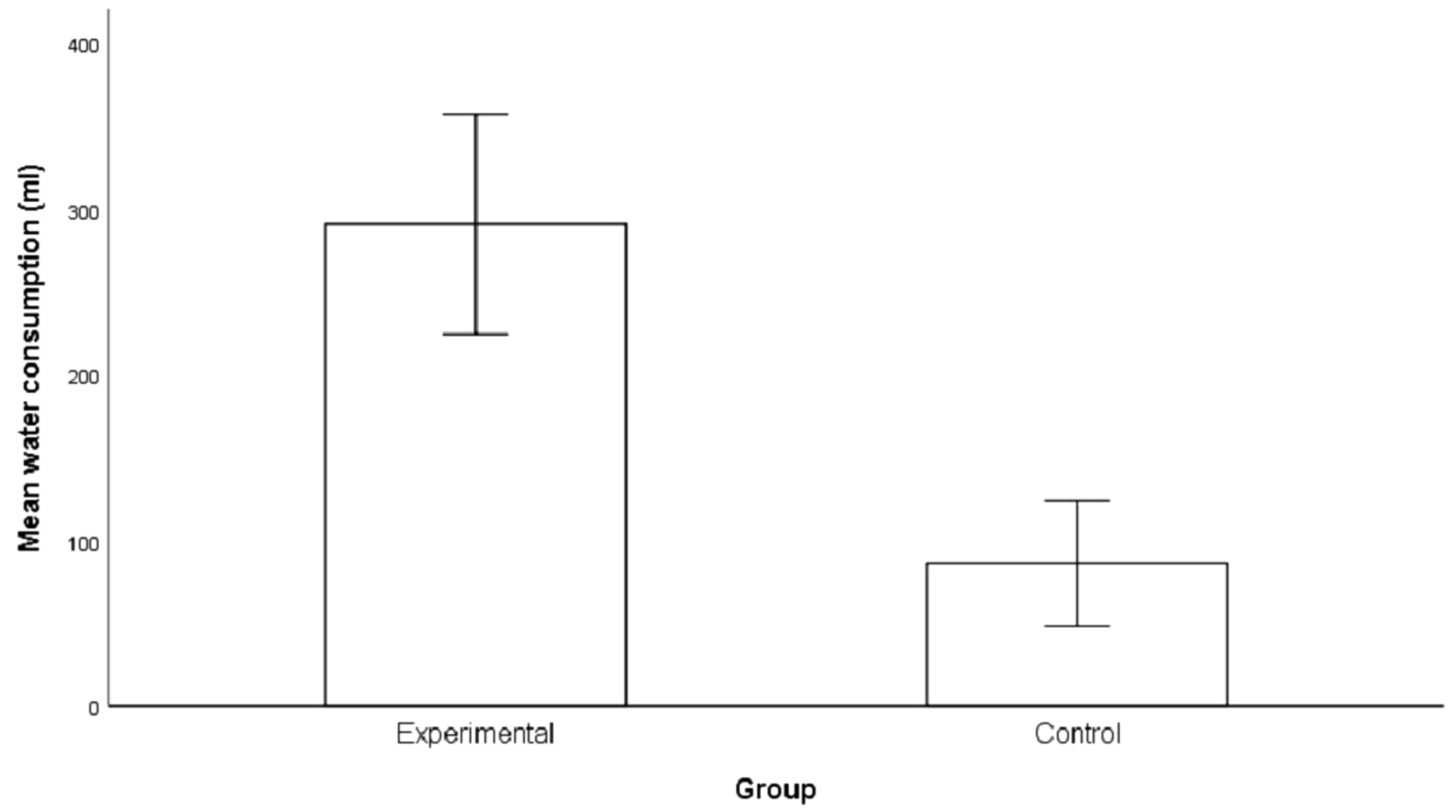
Difference in consumption of water in session 3. The error bars represent the standard errors.

Figure 2 below shows actual differences in the amount of water consumed by participants in the experimental group and control group. Participants in the experimental group consumed on average 291.1 ml of water (SD = 124.7, range = 138–500 ml). Three participants consumed the full amount of water in the 500 ml bottle in the experimental group. Consumption of water in the control group was 86.1 ml on average (SD = 71, range = 0–242 ml). Five participants refused the option to consume water in the control group.

For water consumption, the Shapiro–Wilk tests provided evidence of departure from normality for the experimental group *W*(16) = 0.875, *p* = 0.03. The consumption values for the control group did not show evidence of non-normality *W*(16) = 0.87, *p* = 0.06. Therefore, a Mann–Whitney *U* test revealed significant differences in water consumption between participants in the experimental group (Mdn = 254.00, *n* = 16) and participants in the control group (Mdn = 99.50, *n* = 16), *U* = 10.00, *z* = −4.46, p < 0.001, with a large effect size *r* = 0.79.

## 4. Discussion

The main aim of this study was to contribute to a fuller picture of the relationship between antecedent events that have a motivating function on consumer behaviors, and how to study them by capturing their complexity using multiple methods. In foodservice contexts, deprivation is a relevant antecedent state that individuals experience. To capture the complexity of this relationship between antecedents, consumer behavior, and consequences, we used three experimental sessions to examine visual attention (eye movements), neural responses, choices, and water consumption. The results provide preliminary evidence that the effect of motivating operations can be measured using eye-tracking, EEG, choice, and consumption registration.

Our first question examined whether deprivation would have an EO effect on beverages and evoke a higher fixation count and longer average fixation duration toward the relevant reinforcer. Satiation (having an AO effect) will not show the same impact. A comparison across the two stimulus images (water and salty biscuit) for fixation count and average fixation duration shows that deprivation can affect eye movement. The finding shows that participants in the experimental group had a significantly higher average fixation duration on the stimulus that had an EO effect (water). No differences were present for average fixation duration in the control group. This finding is in line with literature that has demonstrated that deprivation can cause changes in eye-movement behavior especially in terms of the attentional duration metrics toward relevant reinforcers (Castellanos et al., 2009; Hardman et al., 2021). Such research suggests that deprivation might be induced a heightened state of attention toward relevant reinforcers in a visual processing stage related to stimulus recognition and focused attention (Stockburger et al., 2009). For fixation count, the salty biscuit image had higher number of fixations that the water image for the deprivation group. No differences were present for fixation count in the control group. A possible explanation for this result could be due to the stimulus images being presented for a limited time in the experimental sessions. In time-bound conditions, fixation count and average fixation durations seem to have an inverse relationship. This might not be the case in free-viewing conditions. In this research, we can only state that the under the influence of deprivation, only the average fixation duration metric provides some evidence of how the value-altering and behavior-altering effects of motivating operations influence eye-movement behavior.

The second question was to examine whether deprivation would have an EO effect on beverages and evoke relatively greater left frontal activity (approach motivation) toward the relevant reinforcer. Satiation (having an AO effect) will not show the same impact. There was no significant difference in frontal asymmetry activity in the experimental group when viewing the image of the water or the salty biscuit image. There was also no difference present in the control group. However, the results for the experimental group provide us with enough evidence to warrant further replication and extension of this study using the same method. Future studies with a larger sample size would be able would be better equipped to demonstrate differences using frontal EEG asymmetry. Even though prior research has shown that this is a reasonable sample size to demonstrate differences between groups when using neurophysiological tools (e.g., Glaholt and Reingold, 2011; Indira et al., 2012; Telpaz et al., 2015), our results demonstrate otherwise. Consequently, the neurophysiological results of the study depart from previous literature which has reported that frontal EEG asymmetry can be used to measure the motivating function of deprivation (Zinser et al., 1999; Gable and Harmon-Jones, 2008; Harmon-Jones and Gable, 2009).

The third and fourth research questions examined whether deprivation has an EO effect on beverages and evokes (a) choices of the relevant reinforcer and (b) greater consumption of the reinforcer. Satiation (having an AO effect) will not show the same impact on choice and consumption. The empirical results are in accordance with the logic of the concept of motivating operations. Deprivation caused changes in the preferences of participants in the experimental and control groups, supporting previous studies that have examined the influence of motivating operations on choice behavior (Fagerstrøm et al., 2010; Fagerstrøm and Ghinea, 2011). It is interesting to note that the value-altering and behavior-altering effect of motivating operations caused by water deprivation was particularly evident in the consumption of water (the reinforcer). The experimental group consumed almost three times the amount of water compared to the control group. These results demonstrate the EO effect of deprivation and the AO effect of satiation on choice and consumption.

This study makes several important contributions. Due to the current progress in neurophysiological research and greater accessibility to neurophysiological tools, researchers in several fields have shown interest in cross-disciplinary collaboration with neuroscientific research to understand human motivation. This research contributes by complementing existing sources of data, using a multi-method approach, to gain a more comprehensive understanding of antecedent events that have motivating functions on consumer behavior. We measured observable phenomena, namely, behavior and the range of activity on the brain level (Ortu and Cihon, 2019), thus providing objective and reliable insights into consumer behavior (Nevid, 2010; Shaw and Bagozzi, 2018). This research captures the complexity of such interactions by measuring multiple value-altering and behavior-altering effects of motivating operations on consumer behavior.

The findings from the current study contribute to the knowledge by integrating behavioral and neural data for a fuller assessment of behavior (Donahoe, 2017; Ortu and Vaidya, 2017; Hantula, 2018). Such collaboration between research fields will help shed light on complex behavioral phenomena (Ortu and Cihon, 2019; Newland, 2020) by using behavior analysis principles to explain phenomena on neural levels (Hantula, 2019). This is in accordance with current research that studies behaviors that neurophysiological tools can measure. These tools can be used to examine stimuli with reinforcing, punishing, and discriminative functions or as motivating operations that alter the reinforcing or punishing effectiveness of other stimuli (Soto, 2020). Such an expanded repertoire of experimental and analytical tools might permit us to make progress in our understanding of complex behavior (Palmer, 2010; Granerud-Dunvoll et al., 2019; Ortu and Vaidya, 2020). To the best of our knowledge, human participants have not been used when studying the effect of water deprivation from a behavioral perspective, i.e., as a motivating operation. We show the role of motivating operations in controlling human behavior, thereby enabling a richer assessment of behavior (Sundberg, 1993). The benefit of using such a functional account of behavior is that it is predictable and the possibility to control behavior is strong (Pierce and Cheney, 2013). Such an approach should be attractive for researchers and practitioners who focus on consumer motivating functions in retailing, both in physical and online stores.

The sample consisted of 32 participants, in spite of this, the study was able to indicate some differences in a diverse topographies of behavior. However, it would be advisable for future studies to use larger sample sizes. Data collection in this study was conducted in an artificial setting, that is, in a laboratory. The laboratory setting enabled the reduction of artifacts in the collection of neurophysiological data. For this study, data collection was carried out in a controlled experimental setting. Future research could replicate this study in-store with a few more participants to see whether the results differ from laboratory settings. Using a similar multi-method approach, future studies could also examine other relevant motivating operations to test the robustness of the proposed approach. Some research has demonstrated that food-related attentional behavior varies along with different time courses, and is modulated by the type of stimulus and food energy (Luo et al., 2023). Future research can replicate this study and examine the relationship between stimulus type, calorie content, deprivation duration, and visual search behavior. More and more companies are using neurophysiological measures to evaluate and tweak the design of their products (Burkitt, 2009). Our goal was to provide a methodological procedure for management and academia that can remove some of the speculative interpretation of neurophysiological and biometric data. The procedure enables this by framing everything in a behavioral science logic.

## 5. Conclusion

The use of neurophysiological methods to examine different marketing effects is evident through a number of commercial applications by many notable companies. In a similar vein, the procedure outlined in this study can help store owners assess the influence of relevant antecedent events that influence consumers inside the store. Such an approach can be used to evaluate the effectiveness of design elements (like in-store layouts and interface designs of in-store technology) within store settings. Thus, the contribution to practice is a systematic procedure for using multiple neurophysiological methods. The study provides preliminary evidence that situational antecedent events have motivating effects on attention, neural responses, choice, and actual consumption. In turn, these situational factors have an influence on consumer behaviors. This research presents a thought provoking and challenging perspective on behavior science. The combination of multiple methods yields richer insights to examine such complexity compared to using a single method. Our results provide a fuller understanding of antecedent situational effects on consumer behaviors, as well as highlighting the potential application of neuroscientific tools in consumer behavior analysis. Future research could use the methodological procedure of this study using the same design and number of conditions. This will help test the robustness of the multi-method approach that combines behavioral science with neurophysiology to examine the motivating functions of consumer behavior using a larger sample size. Future research can examine other antecedent motivational factors besides water deprivation. Such efforts will help provide more empirical evidence of how motivating operations impact attention, neural activity, choice, and consumption.

## Data availability statement

Upon request, the raw data supporting the conclusions of this article will be made available by the authors, without undue reservation.

## Ethics statement

Ethical review and approval was not required for the study on human participants in accordance with the local legislation and institutional requirements. The patients/participants provided their written informed consent to participate in this study.

## Author contributions

SP, AF, and VS: conceptualization and methodology. SP and AF: validation. SP: formal analysis, investigation, resources, and data curation. SP, AF, VS, and EA: writing – original draft preparation, writing – review, editing, and supervision. All authors contributed to the article and approved the submitted version.

## Funding

This research was funded by Kristiania University College and The Icelandic Centre for Research (RANNIS) (grant number 218235-051).

## Conflict of interest

The authors declare that the research was conducted in the absence of any commercial or financial relationships that could be construed as a potential conflict of interest.

## Publisher’s note

All claims expressed in this article are solely those of the authors and do not necessarily represent those of their affiliated organizations, or those of the publisher, the editors and the reviewers. Any product that may be evaluated in this article, or claim that may be made by its manufacturer, is not guaranteed or endorsed by the publisher.

## Footnotes

^1^ https://imotions.com/

^2^ neurospec.org
